# Diversion and liaison services in England and Wales for mentally disorder offenders – a narrative review

**DOI:** 10.1192/bjo.2021.722

**Published:** 2021-06-18

**Authors:** Patrick McLaughlin

**Affiliations:** National Forensic Mental Health Services

## Abstract

**Aims:**

To critically examine the development of L&D services in England and Wales and critically appraise their evidence base.

**Background:**

High levels of morbidity across the criminal justice pathway are well established. Although the strongest evidence has emerged from prison studies, the court literature also confirms these high levels. In acknowledgment of this, there have been a range of initiatives to improve access to services for mentally ill individuals involved with the criminal justice system. Once such initiative has been the development of court liaison and diversion services (L&D).

**Method:**

Relevant literature was identified through a search of the following databases: PubMed, EMBASE, and PsycINFO. Data were appraised and synthesised to provide a comprehensive overview of the development of L&D services and their evidence base.

**Result:**

The provision of L&D services has increased substantially since their first introduction in England and Wales in 1989. Early L&D services were largely small-scale, unfunded local schemes, and were dependent upon the energy and interest of clinicians who chose to lead in this area. This led to geographical variations in provision and variations in L&D model delivery. The Bradley Report (2009) recommended that a national L&D model be created. The roll-out of a national L&D model meant that half the population of England was covered by 2015, with funding assured for a final wave of L&D services to provide for total population coverage.

Where implemented, L&D services have been shown to lead to increased numbers of local team referrals. They may reduce court adjournments and the overall amount of time spent attending court. There is also some evidence of an association with improved mental health among both adults and young people, with reductions in re-conviction rates amongst the later. There remain deficiencies in the evidence base with regards to the economic impact of L&D services. The majority of economic assessments of L&D services have been performed in the United States with fewer studies in the UK.

**Conclusion:**

Although there is evidence that liaison and diversion can produce benefits, there is a general recognition that a higher standard of evidence is required, including experimental work and assessment of economic impact. L&D services carry a financial burden, but this may be offset by incorporating the value of the health improvements that may be brought in those who might otherwise not have received treatment.

